# Normobaric oxygen therapy in acute ischemic stroke: A pilot study in Indian patients

**DOI:** 10.4103/0972-2327.74203

**Published:** 2010

**Authors:** M. V. Padma, A. Bhasin, R. Bhatia, A. Garg, M. B. Singh, M. Tripathi, K. Prasad

**Affiliations:** Department of Neurology, All India Institute of Medical Sciences, Ansari Nagar, New Delhi 110 029, India; 1 Department of Neuroradiology, All India Institute of Medical Sciences, Ansari Nagar, New Delhi 110 029, India

**Keywords:** Acute ischemic stroke, neuroprotection, normobaric oxygen

## Abstract

**Purpose:**

Clinical and radiological assessment of effects of normobaric high-flow oxygen therapy in patients with acute ischemic stroke (AIS).

**Materials and Methods:**

Patients with anterior circulation ischemic strokes presenting within 12 h of onset, ineligible for intravenous thrombolysis, an National Institute of Health Stroke Scale (NIHSS) score of >4, a mean transit time (MTT) lesion larger than diffusion-weighted image (DWI) (perfusiondiffusion mismatch), and an evidence of cortical hypoperfusion on magnetic resonance imaging (MRI) were included into the trial. Active chronic obstructive pulmonary disease (COPD), requirement of >3/L min oxygen delivery to maintain SaO2 > 95%, rapidly improving neurological deficits, pregnancy, contraindications to MRI, or unstable medical conditions were excluded. The experimental group received humidified oxygen at flow rates of 10 L/min for 12 h. The NIHSS, modified Rankin Score (mRS), Barthel Index (BI) were measured at 0, 1, 7 day of admission and at 3 months follow-up. MRI with DWI/PWI was performed at admission, 24 h later and at 3 months follow-up.

**Results:**

Of 40 patients (mean age = 55.8 years ± 13.2) (range, 26–82), 20 patients were randomized to normobaric oxygen (NBO). The mean NIHSS in NBO and control groups were 14.25 and 12.7 at admission which decreased to 11.6 and 9.5 on the seventh day, and 9.4 and 9.05 at 3 months, respectively. The mean mRS (3.7/3.7) and BI (58.2/53.9) in NBO and control groups improved to 2/2.2 and 73.05/73.8 at the end of 3 months, respectively.

**Conclusions:**

NBO did not improve the clinical scores of stroke outcome in Indian patients with AIS.

## Introduction

The impact of stroke burden in Indian population is tremendous and cannot be overemphasized. Most effective treatments of acute ischemic stroke (AIS) are time-dependent and confine to less than 6 h of stroke onset. World over, modalities to expand the therapeutic time window to enable stroke victims to reach centers where comprehensive stroke treatment can be given is under active investigation. Having a simple technique which can be administered with ease by paramedics or peripheral and secondary care hospitals to “buy time” and arrest the transition of “ischemia” to “infarct”, facilitating transfer to stroke centers for definitive treatment will help improve stroke outcomes. One such potential intervention is normobaric oxygen (NBO) administration.

The results of several recent animal studies have shown that hyperoxia improves pathological, neurobehavioral, and neuroimaging outcomes after stroke.[1–18] NBO therapy administered during ischemia and in the immediate postreperfusion period resulted in a 70% reduction in hemispheric infarct volumes; and could extend the “reperfusion time window” from 1 h to 3 h.[14–16] In a recent pilot trial, NBO-treated patients showed improvement in National Institute of Health Stroke Scale (NIHSS) scores, reduced growth of diffusion-weighted image (DWI) lesion volumes and an increase in the volume of “penumbral” tissue while therapy was being administered.[19,20] In light of these data, NBO might, therefore, be a useful strategy as a neuroprotectant in AIS. A randomized controlled trial was undertaken to study the role of NBO in AIS in Indian patients.

## Materials and Methods

A randomized placebo-controlled study was undertaken with approval of the Institutional Ethics Committee. The inclusion criteria were anterior circulation ischemic stroke presenting within 12 h of stroke onset ineligible for thrombolysis, minimum NIHSS score of ≥4. The exclusion criteria were active chronic obstructive airway disease, patients requiring >2 L/min of oxygen to maintain peripheral arterial oxygen saturation (SaO_2_) > 95%, NIHSS < 4, medically unstable, pregnancy and contraindication to magnetic resonance imaging (MRI). Eligible patients who gave consent were randomized to experimental and control groups.

Block randomization was done with equal allocation of the number in both the groups. NIHSS, modified Rankin Score (mRS), and modified Barthel Index (BI) were recorded after admission, and repeated at the end of 24 h, 7 days, and 3 months. A DWI and perfusion-weighted image (PWI) on MRI was taken at baseline, 24 h and at 3 months wherever feasible. Manual MRI analysis was performed by the neuroradiologist blinded to clinical presentation, treatment group, clinical course, and medications. Stroke volumes were calculated from DWI images. Lesions were outlined on each axial slice using a commercially available image analysis program (ALICE; Perceptive Informatics, Waltham, Mass), to yield total volumes. Postischemic hemorrhage was ascertained on 24-h gradient-echo MRIs. An unblinded clinical investigator monitored patients during therapy.

## Statistical analysis

Statistical analysis was done through SPSS for Windows version 11. For intergroup comparisons, we applied the Student t-test, Mann-Whitney *U*–test, or Fisher exact test for intragroup comparisons, we applied the paired *t*-test or Wilcoxon rank-sum test as appropriate with *P* < 0.05 was considered significant. Repeated measures ANOVA was used to find out the differences intragroup at time points.

## Intervention

The experimental group was given humidified oxygen via a simple face mask at flow rates of 10 L/min for 12 h. The control group was kept under room air or oxygen at 2 L/min via a simple face mask to maintain SaO_2_ ≥ 95%.

## Results

Forty patients (mean age = 55.8 ± 13.2 years) (range, 26–82) completed the study. Twenty patients were randomized in each group. The mean time to randomization for the treatment was 8.5 ± 2.2 h. The mean NIHSS and mRS scores in both the groups remained comparable, both at the base line, at the end of 24 h, 1 week, and at 3 months follow-up. None of the patients reported any adverse effects of the treatment given. The standard treatment of stroke as per guidelines continued in all. Two patients expired in the control group which was unrelated to the treatment (arrhythmia and cardiac arrest and aspiration pneumonia). The deaths were notified to the Ethics Committee.

**Figure 1:**
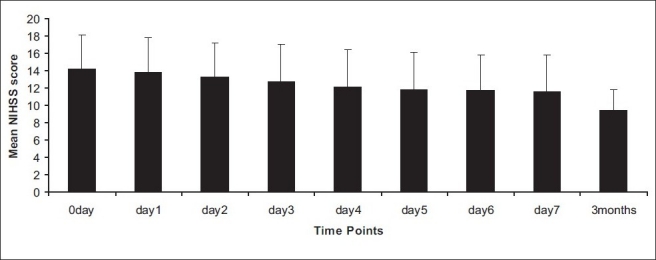
Mean National Institute of Health Stroke Scale score to different time points in experimental group

**Figure 2:**
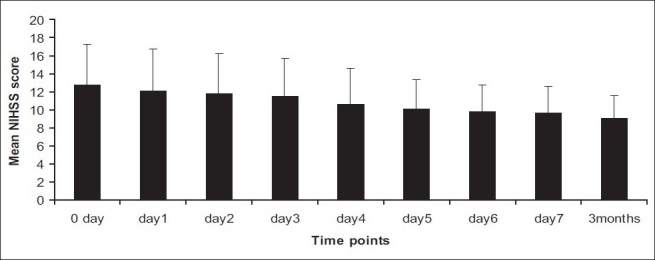
Mean National Institute of Health Stroke Scale score to different time points in control group

There was a significant decrease in NIHSS score from day 0 to day 1 and day 7 to follow-up [Figure 1] in the patients administered with NBO with *P* = 0.002 and *P* = 0.0002, respectively. A similar decrease was also observed in the control group subjects [Figure 2] with *P* = 0.002 and *P* = 0.001, respectively. A trend in improvement was also observed in the mRS and BI in both the groups.

**Figure 3:**
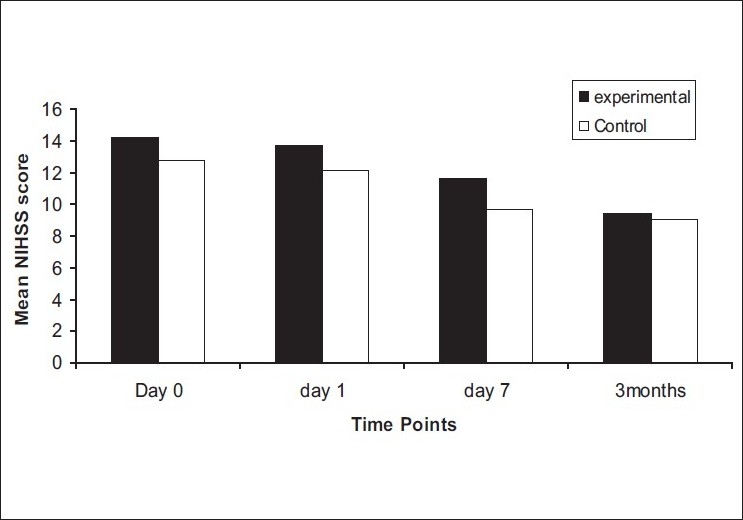
Mean NIHSS score of experimental and control group at day 0, day 1, day 7, and at 3 months (black bar: experimental group, white bar: control group)

**Figure 4:**
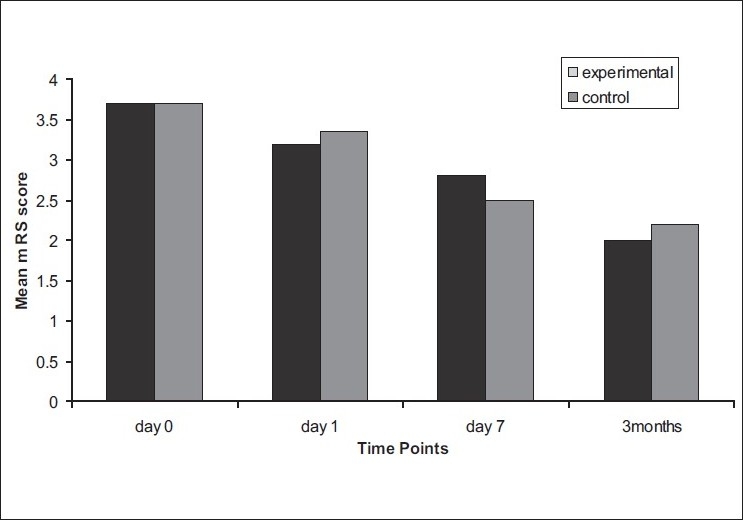
Mean modified Rankin score (mRS) at day 0, day 1, day 7, and at 3 months in experimental and control groups (black bar: experimental group, gray bar: control group)

**Figure 5:**
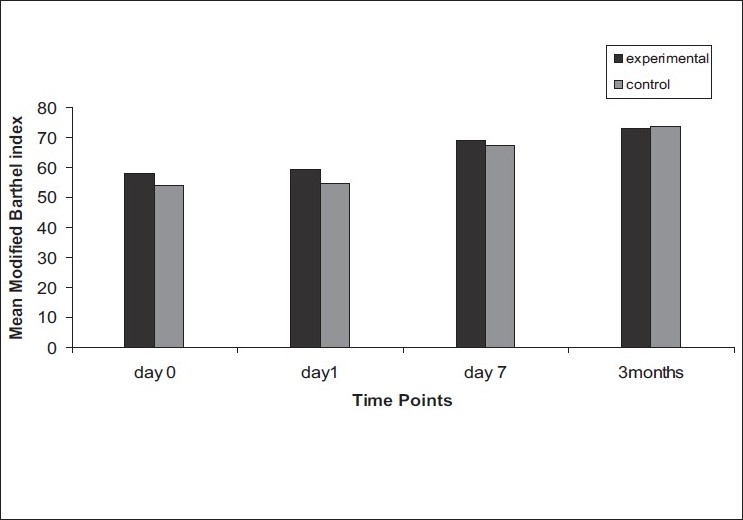
Mean modified Barthel index at 0 day, day 1, day 7, and at 3 months in experimental and control groups (black bar: experimental group, gray bar: control group)

**Figure 6:**
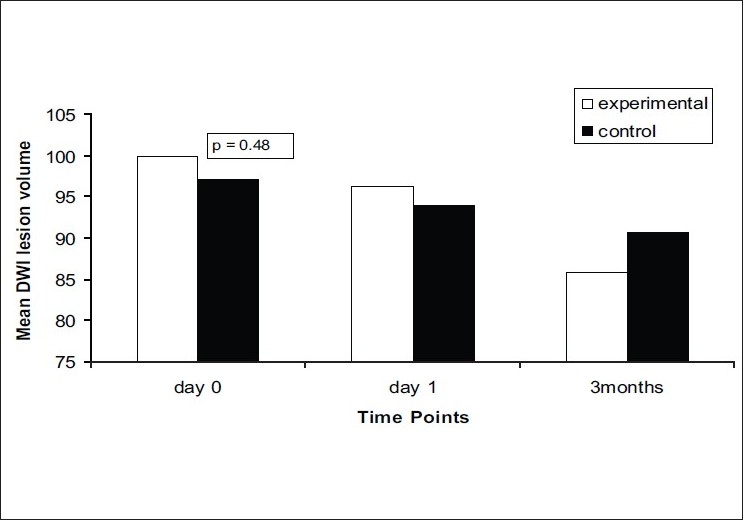
Mean DWI lesion volumes at day 0, day 1, day 7, and at 3 months in experimental and control groups (black bar: experimental group, white bar: control group)

On comparison of the mean of NIHSS between the two groups [Figure 3], no significant difference was observed at day 0, day 1, and at follow-up. At day 7, the mean NIHSS score was higher in the experimental group as compared to control group with a significant *P* = 0.04 (*P* < 0.05). The other two stroke scales, mRS and BI, also did not show any significant improvements at all time points [Figures 4 and 5]. There was no statistically significant difference in the lesion volume between the two groups, although there was a trend toward lesser DWI lesion volume in the experimental group [Figure 6].

## Discussion

Oxygen has multiple beneficial biochemical, molecular, and hemodynamic effects. Hyperoxia might be a useful physiological therapy that slows down the process of infarction and has shown promise in studies of myocardial infarction. Recent studies indicate that brain ptiO_2_ increases linearly with rising concentrations of inspired oxygen and increase nearly fourfold over baseline have been documented in brain trauma patients treated with NBO.[17,20] Since tissue hypoxia is a critical factor in ischemic cell death, there has been a long-standing interest in increasing brain oxygenation to treat stroke.

Hyperbaric oxygen therapy (HBO) has been widely studied because it is the most effective method to increase brain oxygenation.[1–13] HBO in a pressure chamber dramatically increases the oxygen content of blood by physically dissolving oxygen. The increased oxygen content in blood is then released passively in ischemic areas. Numerous animal studies of ischemic stroke have shown HBO therapy at pressures ranging from 1.5 to 3 ATA to be beneficial. However, failure of three small clinical trials combined with the logistic difficulties with HBO chambers, poor patient tolerance, adverse effects of HBO, difficulties of administering HBO with thrombolysis, dampened the enthusiasm of using HBO in AIS.[21–23] A number of human studies in stroke patients (nearly 2000) have indicated that 100% oxygen at pressures of 1.5 ATA is better tolerated than higher pressures by the brain injured by acute ischemia.

An alternative to HBO on account of its limitations will be to use low-pressure oxygen therapy (NBO). Several recent animal studies have shown that short duration NBO can be highly neuroprotective and does not increase oxidative stress, if started early after stoke onset. NBO administered solely during the postreperfusion phase was also found to reduce infarct volumes, which is highly relevant given the theoretical concern of exacerbating oxygen free radical-associated reperfusion injury with supplemental oxygen therapy.[14–18] Further research also that NBO did not significantly worsen blood–brain barrier (BBB) damage, and did not increase the levels of indirect markers of oxidative stress such as matrix metalloproteinase 2 (MMP-2) and MMP-9, hemeoxidase-1 (heat shock protein 32), or protein carbonyl formation at acute or subacute markers of superoxide generation. Additional studies are being conducted to determine whether NBO is safe and effective in models of permanent cerebral ischemia and when used in combination with tPA.

Recently, Singhal and coworkers[19] completed a pilot trial of NBO in which 16 patients with hemispheric ischemic stroke symptoms less than 12 h and diffusion–perfusion mismatch on admission MRI were randomized to 8 h of NBO therapy (45 L/min) or room air. Serial diffusion and perfusion MRI were performed before, during, and after treatment. NBO-treated patients showed improvement in NIHSS scores, reduced growth of DWI lesions, and has previously been documented only with prompt arterial recanalization. There was no radiological or clinical evidence of oxygen toxicity in the small number of patient studies.

In this study, high-flow oxygen therapy started within 12 h of stroke onset did not improve clinical function significantly when compared with those given oxygen at 2 L/min although there was a trend toward improved NIHSS scores. This lack of benefit could be due to delay in initiating the oxygen administration (on an average 6–10 h after stroke onset) and also due to low flow rates (10 L/min) as against 45 L/min used in a previous pilot trial (ref). Our local Ethics Committee did not permit high flow rates. Treatment benefits were not assessed at intervals of 1 h in the first 24 h and the first assessment was only after 24 h. This also could have accounted for a lack of clinical benefit seen since the previous trial documented benefit at 4 h after intervention. However, they did show lasting benefit till 1 week as explained by the significant decrease in the NIHSS score at seventh day [Figure 3].

Out of 20 in experimental group, 11 patients underwent baseline, 24 h and follow-ups MRI. In the control group, nine patients underwent the radiological scanning. Most patients in the acute stage, especially in the first 24 h, were unable to perform the MRI test owing to their neurological condition (they moved in the scanner which made the test difficult). Sedation was avoided as far as possible to avoid altering the sensorium. Clinical assessment was made for all patients included but only a limited number completed the MRI evaluation for DWI lesion volume on account of the above limitation. Similarly, PET scans were not possible within the given period of time due to nonavailability of the dye, patient’s neurological status, and some other practical considerations (limited research time on the PET machine, nonfunctionality of the machine).

The mechanisms of NBO’s effects are not clearly understood. Preliminary data from a subset of patients enrolled in the NBO pilot clinical trial who underwent detailed MR spectroscopy show that NBO improves brain lactate levels, presumably by restoring aerobic metabolism within ischemic brain regions. Using *in vivo* electron paramagnetic resonance oximetry, Liu *et al*.[17] have shown that NBO significantly increases ptiO_2_ in “penumbral” brain region. While NBO does not raise brain tissue oxygenation to the same degree as HBO, it is conceivable that even small increments are adequate to overcome ischemic thresholds. Experimental studies with HBO have shown that supplemental oxygen favorably alters the levels of glutamate, lactate, bcl-2, manganese superoxide dismutase, cyclooxygenase-2, and inhibits cell-death mechanisms such as apoptosis. It is likely that NBO has similar biochemical and molecular effects.[17]

Further studies are needed to validate these preliminary results. Perhaps these need to be conducted at the prehospital setting and as an adjunctive therapy with thrombolysis. Although this was a negative study, potential advantages of this intervention cannot be underestimated, especially in developing countries such as India, where cost-effective strategies are of paramount importance.
